# Classification and Lateralization of Temporal Lobe Epilepsies with and without Hippocampal Atrophy Based on Whole-Brain Automatic MRI Segmentation

**DOI:** 10.1371/journal.pone.0033096

**Published:** 2012-04-16

**Authors:** Shiva Keihaninejad, Rolf A. Heckemann, Ioannis S. Gousias, Joseph V. Hajnal, John S. Duncan, Paul Aljabar, Daniel Rueckert, Alexander Hammers

**Affiliations:** 1 Division of Experimental Medicine, Centre for Neuroscience, Faculty of Medicine, Imperial College London, United Kingdom; 2 Neurodis Foundation,CERMEP – Imagerie du Vivant, Lyon, France; 3 Imaging Sciences Department, MRC Clinical Sciences Centre, Imperial College London, United Kingdom; 4 Department of Clinical and Experimental Epilepsy, UCL Institute of Neurology, Queen Square, London, and National Society for Epilepsy MRI Unit,Chalfont St Peter, United Kingdom; 5 Department of Computing, Imperial College London, United Kingdom; Beijing Normal University, Beijing, China

## Abstract

Brain images contain information suitable for automatically sorting subjects into categories such as healthy controls and patients. We sought to identify morphometric criteria for distinguishing controls (n = 28) from patients with unilateral temporal lobe epilepsy (TLE), 60 with and 20 without hippocampal atrophy (TLE-HA and TLE-N, respectively), and for determining the presumed side of seizure onset. The framework employs multi-atlas segmentation to estimate the volumes of 83 brain structures. A kernel-based separability criterion was then used to identify structures whose volumes discriminate between the groups. Next, we applied support vector machines (SVM) to the selected set for classification on the basis of volumes. We also computed pairwise similarities between all subjects and used spectral analysis to convert these into per-subject features. SVM was again applied to these feature data. After training on a subgroup, all TLE-HA patients were correctly distinguished from controls, achieving an accuracy of 96 ± 2% in both classification schemes. For TLE-N patients, the accuracy was 86 ± 2% based on structural volumes and 91 ± 3% using spectral analysis. Structures discriminating between patients and controls were mainly localized ipsilaterally to the presumed seizure focus. For the TLE-HA group, they were mainly in the temporal lobe; for the TLE-N group they included orbitofrontal regions, as well as the ipsilateral substantia nigra. Correct lateralization of the presumed seizure onset zone was achieved using hippocampi and parahippocampal gyri in all TLE-HA patients using either classification scheme; in the TLE-N patients, lateralization was accurate based on structural volumes in 86 ± 4%, and in 94 ± 4% with the spectral analysis approach. Unilateral TLE has imaging features that can be identified automatically, even when they are invisible to human experts. Such morphometric image features may serve as classification and lateralization criteria. The technique also detects unsuspected distinguishing features like the substantia nigra, warranting further study.

## Introduction

Neurological diseases are frequently characterized by specific pathomorphological changes that can be observed on magnetic resonance (MR) images as localized variations in signal intensity or as changes in the shape and size of individual brain structures. Temporal lobe epilepsy (TLE) is the most common type of epilepsy requiring surgical treatment [1]. Distinguishing the pathological abnormalities underlying TLE is a desirable clinical capability, as patients with hippocampal sclerosis (HS) have a 60% chance of becoming seizure free with surgery [1], [2].

HS is the most commonly detected abnormality in patients with medial temporal lobe epilepsy (TLE with hippocampal atrophy, TLE-HA), observed in around 70% of patients with “non-lesional” TLE [3]. HS can be detected on MR images and is characterized by volume loss in T1-weighted images [4]–[8] in combination with increased signal on T2-weighted [9], [10] and FLAIR images [11], [12]. Aside from the hippocampus, there are other structures in the brain which are affected in TLE-HA. Volume reductions have also been reported for the thalamus [13], [14], caudate nucleus and putamen [15], and amygdala [16]. This growing body of evidence shows that TLE-HA is not merely a focal disease of the hippocampus, but a systemic disease that affects brain structures both close to and distant from the seizure focus [17]. Many of the studies cited above were carried out by manually delineating selected brain structures. This labour-intensive procedure necessitates a selective approach, which explains why only a small number of structures have been evaluated so far. To reduce the workload and increase reproducibility (if not necessarily accuracy), several studies have developed automated or semi-automated methods, using for example seedpoints or bounding boxes [18]–[20], voxel-based morphometry (VBM) [21], [22], shape models [23] or atlas-based segmentation [5], [24], [25].

VBM is a largely automated whole-brain technique for characterizing structural brain differences *in vivo* [26] and the technique has frequently been used to study patients with epilepsy [21], [27], [28].

For detecting focal pathology, VBM and optimised VBM tend to be insufficiently sensitive, especially when pathomorphological changes are relatively subtle, as is the case in hippocampal sclerosis [22], [29], [30]. Atlas propagation is a method that can be used as a segmentation method in its own right [25], [31] or as a way of providing prior information for a further segmentation step [32], [33]. Multi-atlas label propagation has been shown to be a reliable approach for automated detection of hippocampal sclerosis in individual patients with TLE [5].

It is estimated that in at least 30% of TLE patients, visual and volumetric evidence of HS as well as abnormalities of T2 relaxation time are absent. We refer to this condition as TLE-N (MR imaging negative, [34], [35]). It is possible that some of these patients have normal hippocampi, and others may have subtle hippocampal damage that can not be detected by visual review of *in vivo* structural MRI [36]. Various abnormalities have been described in TLE-N mostly in studies targeting the temporal lobe only using variety of techniques: regions of interest [37], VBM [27] or a combination of region-based and voxel-based methods [38], magnetic resonance spectroscopy [39], positron emission tomography (PET) with FDG [40], PET with flumazenil (a GABA*_A_* receptor ligand, [41]) and PET with 5-HT1*_A_* ligands [42], SPECT [43], [44], T2-weighted images using voxel-based relaxometry and interictal as well as ictal electroencephalography (EEG) [45]. In contrast to MRI, they are not part of the routine clinical workup. Recently, there has been interest in MR brain image classification using pattern recognition methods based on feature extraction, dimensionality reduction, and classification [46], [47]. Machine-learning techniques such as support vector machines (SVMs) are used with the aim to classify structural or functional brain images into two groups (e.g. male/female or patient/control, [38], [46], [48]). In brief, SVM is a tool that is trained with a sample of data classified according to a gold standard. These data are mapped into a higher-dimensional space where a linear separation is sought. Support vectors are identified in this new space as the datapoints in each class lying closest to the best separating linear boundary (hyperplane) between the classes. New datasets can subsequently be mapped into the same space and classified depending on which side of the hyperplane they fall. Advantages of this method are the automatic selection of training examples that are most informative for the classification; good scalability to large numbers of possible classifying features; and the possibility of training classifiers based on small training sets. Classification methods for the distinction of different TLE patient classes from one another and controls, but in particular for the lateralization of the epileptogenic side in cryptogenic TLE-N, based on standard MRI, would be highly desirable. Automatic classification attempts in other diseases like Alzheimer’s disease (AD) have largely been voxel-based; as outlined above, standard voxel-based detection does not perform well in the case of HA.

We have previously shown that the predecessor method for multi-atlas propagation and label fusion [25] was able to correctly identify hippocampal atrophy as one element of unilateral HS [5], and Ð importantly Ð correctly identify contralateral hippocampi as being of normal volume. In this work we use MAPER (Òmulti-atlas propagation with enhanced registrationÓ, [49]), an automatic brain segmentation method based on multiple atlases [50] that is better suited to the automatical segmentation of pathological MRIs [49], [51] and was previously shown to work very well in normal human brain images and patients with TLE and AD [49]. A structure selection technique using a kernel based class separability criterion is performed to identify the structures that most readily discriminate between pairs of subject groups (patient/control; TLE-HA/TLE-N; left/right TLE). In this study the term “structure selection” is equivalent to “feature selection” in the context of pattern recognition, where the features are the structural volumes adjusted for intracranial volume (ICV). Once the most relevant structures have been ranked and selected, classification is completed using a suitable machine learning method. Two classification procedures based on selected structural volumes and morphological similarity are used for classification. In the first procedure, a supervised classification method (SVM) is applied to the structural volumes adjusted for ICV. The accuracy of this classification scheme is dependent on the group separability provided by each structure’s volume. We demonstrate that, as expected the accuracy of this classification scheme decreases when control and patient classes are not well separated by their structural volumes. This problem most affected the separation of the TLE-N and control groups. To address it, we derived pairwise measures of morphological similarity between subjects using the differences in volume between corresponding selected structures.

## Materials and Methods

### Experiment Overview

**Figure 1:**
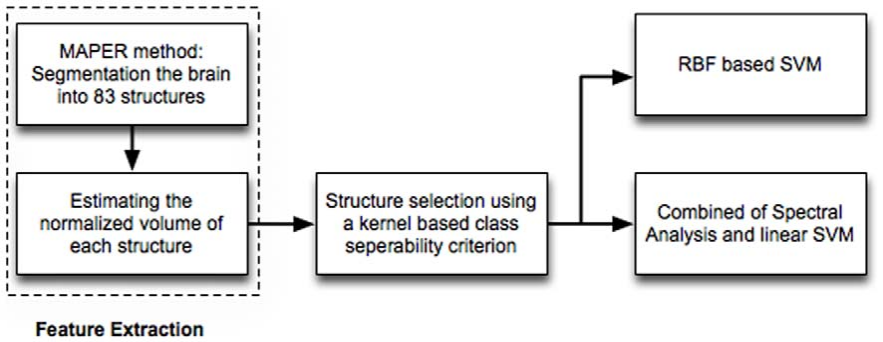
The analysis pipeline of the proposed classification scheme. MAPER: multi-atlas propagation with enhanced registration; RBF: radial basis function; SVM: support vector machine.

An overview of the three-stage analysis is shown in Figure 1. To assess the classification accuracy of the proposed methods, five experiments were performed:

- Experiment 1 – TLE-HA vs. control: classification of TLE-HA subjects versus control subjects.
- Experiment 2 – TLE-HA_R vs. TLE-HA_L: lateralization of the site of seizure onset in the TLE-HA group.
- Experiment 3 – TLE-HA vs. TLE-N: classification of TLE-HA subjects versus TLE-N subjects.
- Experiment 4 – TLE-N vs. control: classification of TLE-N subjects versus control subjects.
- Experiment 5 – TLE-N_R vs. TLE-N_L: lateralization of the site of seizure onset in the TLE-N group.

Experiments #1, 2, 3, and 4 are designed to assess the performance of the method. Clinically, experiment #1 corresponds to a clinically important screening situation (TLE-HA patients are managed differently from those without HA, see discussion in [5], and experiment #5 addresses the clinically important question of lateralization in the absence of MRI changes.

### Subjects

Demographic features of the population, details of image acquisition and clinical characteristics are summarized in Table 1. The patient group consisted of 80 subjects with clinical and neurophysiological characteristics of TLE, whose MR images and clinical details were obtained from the database of the National Society for Epilepsy. The database record contained a consensus diagnosis based on visual assessment of the MR images by two experienced neuroradiologists with a special interest in epileptology. Sets of T1-weighted images from five groups were used in this study. Sets of T1-weighted images from five groups were used in this study.

**Table 1:** Demographic features of the group of patients with temporal lobe epilepsy (TLE), controls and atlas images. Median (range) of Age and Age at onset is reported.

No	Group 1 TLE-HA	Group 2 TLE-N	Group 3 Controls	Group 4 Atlases	Group 5 TLE-HA*
	60	20	28	30	9
Sex (female)	29	9	14	15	5
Age (years)	39 (19–66)	38 (23–53)	31 (19–55)	31 (20–54)	38 (22–49)
Right	27	9	–	–	5
Left	33	11	–	–	4
Field strength (T)	3	3	3	1.5	1.5
Age at onset	7.5 (0.5–31)	14.5 (1–32)	NA	NA	5 (1–23)
Febrile seizures	22	1	NA	NA	5
Family history of epilepsy	19†	4‡	NA	NA	–**

TLE-HA: TLE patients with visually diagnosed hippocampal atrophy, TLE-N: TLE patients with normal MRI, TLE-HA.

* : TLE patients with visually diagnosed hippocampal atrophy from [5].

† : information missing for 9 subjects.

‡ : information missing for 1 subject.

** : information missing for this group.

Group 1∶60 patients had visually detected unilateral HA (TLE-HA, median age of 39 years, mean age ± SD 39 ± 12 years, 29 women). All patients had unilateral HS on expert visual MRI assessment, including hippocampal quantification (volume loss on T1-weighted and intensity change on T2-relaxometry) when judged necessary by the two neuroradiologists [52], [53]. HA was always ipsilateral to the site of seizure origin as determined by combinations of history, semiology, interictal and ictal EEG and neuropsychological assessment. 27 patients had right HA, and 33 had left HA.

Group 2∶20 patients had normal MRI scans (TLE-N, median age of 38 years, 36 ± 10 years, 9 women).

Group 3∶28 healthy individuals (median age of 31 years, 32 ± 11 years, 14 women), scanned on the same 3T scanner as the patients, were included in this study.

Group 4 (Atlases): 30 subjects (median age of 31 years, 31 ± 8 years, 15 women) whose MRIs had been manually segmented into 83 anatomical structures [50], [54].

Group 5: To test the ability of the proposed method of distinguishing patients with TLE from controls, nine images of subjects affected by TLE-HA were considered as the test group. T1-weighted MRIs of this patient group had been acquired at the National Society for Epilepsy in Chalfont St Peter, United Kingdom. Acquisition and demographical details have been previously published [5]. Demographics are summarised in Table 1. Acquisition details were identical to those used for the atlas images.

T1-weighted atlas images and Group 5 were acquired on a 1.5 Tesla GE Signa Echospeed scanner at the National Society for Epilepsy. A coronal T1-weighted 3D volume was obtained using an inversion recovery prepared fast spoiled gradient recall sequence (GE), TE/TR/NEX 4.2 ms (fat and water in phase)/15.5 ms/1, time of inversion (TI) 450 ms, flip angle 20°, yielding 124 slices of 1.5 mm thickness with a field of view of 18×24 cm for a 192×256 matrix, covering the whole brain with voxel sizes of 0.9375×0.9375×1.5 mm. Images were resliced to create isotropic voxels of 0.9375×0.9375×0.9375 mm^3^ using windowed sinc interpolation to preserve the native resolution.

T1-weighted images for patients and control subjects were collected on a 3T GE scanner using FSPGR, TE/TR/NEX 3 ms/8 ms/1, time of inversion (TI) 450 ms, flip angle 20°, yielding 170 slices of 1.1 mm thickness with a field of view of 18×24 cm for a 256×256 matrix, covering the whole brain with reconstructed voxel sizes of 0.9375×0.9375×1.1 mm^3^.

The groups in the various experiments did not differ significantly in terms of gender; there were some small age differences in Experiment 1 (see Results Section). As expected, there was a difference between TLE-HA and TLE-N in terms of age at onset (7.5 years vs 14.5 years, Mann-Whitney U test *p* < 0.05).

Approval for scanning the controls had been obtained from the Joint Ethics Committee of The Institute of Neurology and the NHNN (National Hospital for Neurology and Neurosurgery), and written informed consent obtained prior to scanning. Post-processing of anonymised scan data that had been acquired for clinical purposes did not require individual consent from the individuals who had been scanned.

### Automatic Segmentation

The MAPER was used to automatically delineate 83 regions of interest (ROI) in every brain. Twenty of these paired structures are located in the temporal lobes; 24 in the frontal lobes; six in the parietal lobes; six in the occipital lobes; three in the posterior fossa; six in the insula and cingulate gyri. Thirteen are central structures and five ventricular regions. A full list of ROIs is available in [54] and in the Supporting information (Text S1).

**Figure 2:**
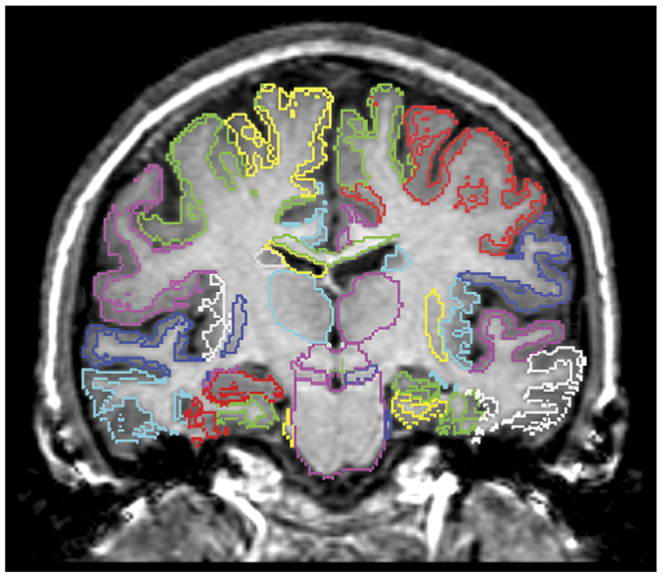
Example segmentation result using MAPER. Coronal section through the T1-weighted 3D MR image of a subject with left hippocampal sclerosis. The left of the subject is shown on the right of the image. Note the clear difference between the atrophic left and normal sized right hippocampus. Other volumetric differences relevant for automatic classification are invisible on visual inspection.

MR images were preprocessed using tools from the FSL suite (Version 4.1, [55]). Preprocessing of the atlas, control and TLE sets consisted of brain extraction and bias correction using “BET” and “FAST”. The parameters used in the brain extraction step were tuned for each dataset, and those which resulted in the best strip (as judged visually removal of scalp, skull, CSF and dura with preservation of brain tissue) were used. Tissue probability maps for each subject for each of the main classes: grey matter (GM), white matter (WM) and cerebrospinal fluid (CSF) were generated using FSL FAST. The tissue class maps were treated as inputs to a multichannel registration. Atlas and target images were aligned using rigid, affine and coarse non-rigid (20 mm control point spacing) registration using a free-form deformation model based on B-splines [56] and optimizing cross-correlation over all three tissues (channels) simultaneously. The resulting transformation was used as the starting point for a more detailed non-rigid registration of the MR intensity images using normalized mutual information as the similarity criterion with the same parameters as described in [25]. Non-rigid registration is performed at control point spacings of 10 mm, 5 mm and 2.5 mm. These steps are carried out using each of the 30 atlases in turn, resulting in 30 segmentations per target brain, which are subsequently combined using vote-rule decision fusion [57]. Figure 2 shows the segmentation results on a TLE-HA subject.

**Figure 3:**
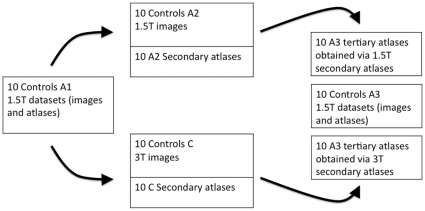
The flowchart of the experiments on assessing the potential bias resulting from the difference in field strength between atlas images and segmentation targets. A1, A2 and A3: groups of ten subjects from the 30 atlas datasets scanned at 1.5T. group C: ten randomly selected 3T images from the control set. Middle column top row: A1 datasets were used to anatomically segment A2 images with MAPER, resulting in automatically labeled images (A2*_secondary_*). These secondary atlas datasets were then used to segment the A3 images with MAPER. Middle column bottom row: A1 datasets used to segment group C with MAPER. The resulting ten secondarily labeled group C datasets were then used to anatomically segment the A3 images with MAPER. Last column: three sets of anatomical segmentations for A3 images: two automatically generated either via 1.5T or 3T secondary atlases, and one manual gold standard segmentation.

Atlases of the whole brain had been manually drawn on 1.5T MR images, whereas all the patients and controls studied had been scanned at 3T. This difference in field strength might bias the segmentation results. We performed a set of experiments (Figure 3) with intermediate target images acquired either at 1.5T or 3T to assess the influence of field strength for segmentation accuracy:

Firstly, the 30 atlas datasets scanned at 1.5T were randomly divided into three groups of ten (A1, A2 and A3). A1 datasets were used to anatomically segment A2 images with MAPER, resulting in automatically labeled images (A2*_secondary_*). These secondary atlas datasets were then used to segment the A3 images with MAPER.

Secondly, A1 datasets were used to anatomically segment ten randomly selected 3T images from the control set (group C) with MAPER. The resulting ten secondarily labeled group C datasets were then used to anatomically segment the A3 images with MAPER.

At the end of this procedure, there were three sets of anatomical segmentations for A3 images: two automatically generated either via 1.5T or 3T secondary atlases, and one manual gold standard segmentation. The region-by-region overlap of the two automatically generated anatomical segmentations with the manual A3 segmentations was then assessed.

Hippocampal volumes and other brain structural measurements may vary with head size, thus head size is a confound for between-subject comparisons. Normalization by intracranial volume reduced variability in volume measurements of nearly all brain regions to a greater extent than did normalization by other methods [58]. As a correction factor for interindividual variations of head size, the total ICV was measured therefore using an automated and robust method, Reverse MNI Brain Mask (RBM, [59]), where a standard mask in MNI space derived from tissue probability maps is warped to each image in native space using the inverse of the normalizing transformation. To identify each region’s grey matter portion, probabilistic GM maps were thresholded at 50% probability for each subject. Voxels above the threshold are counted for estimating the volume of grey matter within the identified structures. Structures that either contain no GM (ventricles, corpus callosum) or contain GM that is typically misclassified as having ≤ 50% probability of GM with current tissue segmentation algorithms (caudate nucleus, nucleus accumbens, pallidum, putamen, substantia nigra, thalamus and brainstem) were excluded from this masking procedure. All volume measurements (18 full structures plus 65 grey-matter portions) were normalized by ICV.

### Structure Selection

We use the set of 83 structural volumes from each MR image as a sparse description of the brain morphology of each subject. Some structures will be affected by TLE to a lesser extent, or not at all, and will thus be less useful for classification. We therefore sought to identify the most effective structures in order to obtain a suitable final classifier. To achieve this, we used a class separability criterion to rank the structures. The higher the value of the class separability criterion of a structure, the more the structure contributes to discriminating the two classes.

In this study we employed a kernel-based class separability criterion as proposed in [60] using the procedure described in Supporting Information (Text S2). The advantage of this criterion over more conventional criteria such as the Bhattacharyya distance, Kullback-Leibler divergence, and Matusita distance [61] is that no assumption is made regarding the conditional probability densities of features (volumes of structures). Furthermore, it is applicable to linearly non-separable data and is informative when a class contains few samples. For selecting *D* structures from *M*  =  83, *D* ≤ *M*, we used the *Best Individual N (BIN)* technique [60]. In BIN, the class separability criterion (see Eq. 4 in Text S2) is individually applied to each of the features and those with the largest values are selected.

### Spectral Clustering Approach for Classification

The morphological similarity of corresponding structures between pairs of subjects can be used for group classification. Spectral analysis is a technique which converts pairwise measures of similarity between subjects into per-subject features to which standard classification or clustering techniques can be applied.

For brevity, we omit a full description of spectral clustering; details are available in Supporting Information (Text S3) and more general description in [62]. At a high level, spectral clustering employs the following four steps:

- Construct a complete, undirected graph where the nodes are subjects and the edges are weighted by pairwise morphological similarity between the subjects.
- Define the Laplacian matrix of the graph and generate feature vectors from the eigenvectors of this matrix.
- Cluster the features using conventional classification algorithms to assign group membership to each subject.

In this work, we used the volumetric difference described by the Gaussian similarity function 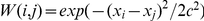, where *c* is a constant of value 2 as obtained empirically in [63] and variables 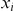 and 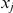 correspond to the normalized volumes of a particular structure in subjects *i* and *j*, respectively. The volumes of corresponding selected structures over *N* subjects were transformed to z-scores, 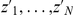 by subtracting the mean and dividing by the standard deviation. For a general description on this use of the Gaussian form as a neighbourhood or similarity function, see [62], [64], where it is described as a heat kernel. Separate Laplacian matrices 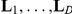 are constructed for the *D* structures identified by structure selection. The feature data from separate Laplacian matrices are then combined to create the *N* × *kD* feature matrix, with each row corresponding to a feature extracted for a subject. Since ours is considered a two class problem, we chose *k*  =  2 as suggested in [62]. We then employed a linear SVM model for learning to classify within the constructed feature space.

### SVM-Based Classification

A support vector machine (SVM) is an example of a supervised binary classification method [65].

The key concept of SVM is the use of hyperplanes to define decision boundaries separating between data points of different classes. SVMs are able to handle both simple, linear, classification tasks, as well as more complex, i.e. nonlinear, classification problems. The idea behind SVMs is to map the original data points from the input space to a high-dimensional, feature space such that the classification problem becomes simpler in the feature space. The mapping is done by a suitably chosen kernel function. The use of SVM involves two basic steps, namely training and testing. Training an SVM involves feeding labelled data to the SVM, thus forming a finite training set. The separation learned from the training data can then be applied to the testing data.

SVMs were used in two ways in this work: first, a nonlinear SVM using a radial basis function (RBF) was applied to the ranked selected structural volumes directly. Second, a linear SVM was applied to feature data derived from spectral analysis of similarities.

For each experiment (TLE-HA vs. control, TLE-N vs. control and TLE-HA vs. TLE-N) two classifiers were trained. The posterior probabilities were computed using i) the classifier trained by the selected structures of TLE-HA_L and control subjects and ii) the classifier trained by the selected structures of TLE-HA_R and control subjects. A corresponding approach was used for classifying TLE-N vs control subjects and TLE-HA vs. TLE-N.

There are two concerns in using SVM. First, the parameter of the RBF kernel and slack variable are not known beforehand, consequently a model selection or a parameter search process must be performed [66]. The goal is to set the parameters such that the classifier can accurately predict unknown data (i.e. testing data). Second, there is no prior information about the optimal number of structures that grants the best average correct classification rate. A common way to identify the optimal parameters and number of structures is cross-validation. Therefore, we set a grid search on the RBF kernel parameter, the slack variable and the number of structures using leave-one-out cross-validation. We used the SVM algorithm implemented by the LibSVM package, an integrated software for support vector classification (www.csie.ntu.edu.tw/~cjlin/libsvm).

### Statistical Analysis

Pearson correlation coefficients were used to evaluate relationships between hippocampal volumes and ICV. The significance level for all analyses was set at *p* < 0.05. Means were compared with the Student’s t-test, and medians were compared with the Mann-Whitney U test. The data were analyzed using SPSS Version 16 for Microsoft Windows (SPSS Inc., Chicago, IL, USA).

To evaluate the performance of different classification methods, we used a 10-fold cross-validation strategy. The classification accuracy (for measuring the proportion of subjects correctly classified among the whole population), as well as the sensitivity and the specificity were computed. The entire set of subjects were partitioned into 10 equal subsets. At each iteration, the subject samples within one subset were selected as the testing samples, and all remaining subject samples (the other 9 subsets) were used for training the classifier. This process was repeated 10 times independently to avoid possible bias resulting from random differences between the testing and the training set. The average accuracy, sensitivity and specificity of classification resulting from the 10 × 10 runs are reported. To evaluate the performance of classifiers in Experiment 5, which contains 20 subjects, five-fold cross-validation was used. Five-fold cross-validation randomly divided the data into five groups of approximately equal size. Here four groups were used as training set, and one group was used as testing set. This was done five times, each time rotating the data in the training and testing sets, resulting in five performance results computed on the individual groups, which were averaged. The cross-validation was repeated ten times, with different composition of the cross-validation groups.

The statistical significance of the classification rates was estimated using permutation testing. This assesses the statistical significance of the classifier by estimating the probability of obtaining the observed classification performance under the null hypothesis that the classifier cannot learn to predict labels based on the given training set [67]. In this approach, the clinical labels for the subjects are permuted and a full leave-one-out cross validation is carried out using a classifier based on the top ranked structures. The classification rate associated with the permutation is then calculated. The permutation procedure was repeated 10,000 times to estimate the distribution of classification rates. This distribution was then used to estimate the significance of the classification rate observed with the original unpermuted labels. For each experiment a separate permutation test was carried out.

## Results

To investigate the effect of age on regional volume and consequently on the classification results, the age differences between groups in each experiment were studied using the Mann-Whitney U test. There was a small but significant age difference for Experiment 1 (TLE-HA vs. control) when considering controls (median 31 years) and all subjects in the TLE-HA group (median age 39, *p*  =  0.036). However, this age difference was not significant between controls and either TLE-HA R (*p*  =  0.059) or TLE-HA L (*p*  =  0.069). There were no significant age differences between any of the groups in Experiments 2–5 (*p* 0.1–0.8).

The experiments on assessing the potential bias resulting from the difference in field strength between atlas images and segmentation targets showed the overlaps based on the atlas (A2) as intermediates are slightly larger than overlap based on the 3T controls (C) as intermediates (1.05% ± 4.6, mean ± SD).

The mean and standard deviation of the intracranial volume (*p* value as compared with controls), in *cm* ^3^ for the control group was 1483±160. For the TLE-HA group it was 1387±128 (*p* < 0.05), and for the TLE-N group 1423±150 (*p* > 0.1).

Hippocampal volumes were correlated with ICV in all subjects, and a significant correlation was present in all subgroups (TLE-HA: 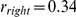, 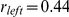, TLE-N: 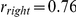, 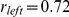, control: 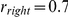, 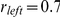, all 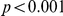).

**Figure 4:**
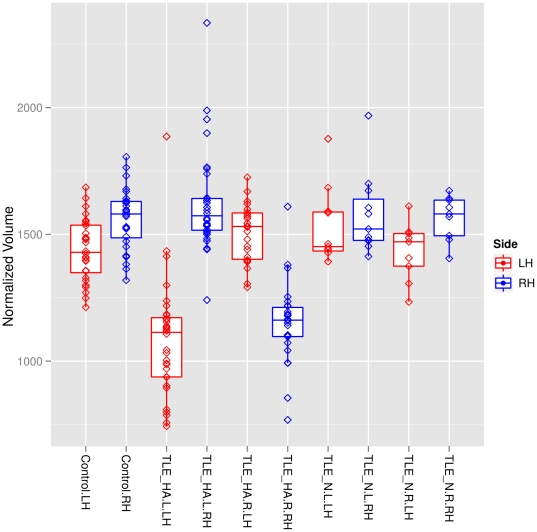
Hippocampal volumes in patients and controls. Horizontal lines show the medians, boxes indicate interquartile ranges, whiskers show the minimal and maximal values inside the main data, and lozenges show individual values. Blue, right hippocampi; red, left hippocampi. TLE-HA, TLE with hippocampal atrophy; TLE-N; TLE with normal MRI on visual inspection. Suffixes _L and _R denote left and right sided seizure focus, respectively.

We did not observe a correlation between hippocampal volumes and age, probably because the age range was narrow in all groups. The correlation of the classification-relevant brain structures with age for patients and controls is reported in the Supporting information (Text S4). There was no significant effect of gender on ICV-adjusted structural volumes (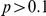). Figure 4 shows the normalized, grey-matter masked ipsilateral and contralateral hippocampal volumes of the TLE-HA, TLE-N and control groups. The coefficients of variation for all regions and groups is available in the Supporting information (Text S5).

### Structure Selection

Table 2 and 3 show the top-ranked structures after applying the structure selection method, as well as the ability of each individual structure to separate the TLE-HA and TLE-N group from the control group assessed on a leave-one-out basis using SVM-RBF. The effect of combining these top-ranked structures is also shown. By introducing other structures (e.g. amygdala, anterior orbital gyrus, anterior temporal lobe lateral part), all TLE-HA subjects with left sided seizure focus can be distinguished from the control subjects. All TLE-HA subjects with a right sided seizure focus are separated from controls by including parahippocampal gyrus, thalamus, and anterior orbital gyrus. Table 2 shows that the discrimination ability of the individual structures ipsilateral to the epileptogenic focus is smaller than that of the hippocampus in both groups and aggregating top-ranked structures ipsilateral to the epileptogenic focus yielded 100% sensitivity. The automatically selected structures in the TLE-N group ( Table 3 ) are mainly ipsilateral to the presumed seizure focus, and largely orbitofronto-temporal.

**Table 2:** Top structures ranked by ability to distinguish TLE-HA patients from controls.

Structure	TLE-HA_L Individual Sensitivity(%)	Combined Sensitivity(%)	Structure	TLE-HA_R Individual Sensitivity(%)	Combined Sensitivity(%)
Hippocampus*_L_*	93	–	Hippocampus*_R_*	92	–
Amygdala*_L_*	75	96	Parahippoc G*_R_*	74	92
Ant orbital G*_L_*	75	96	Thalamus*_R_*	74	96
Ant tmp L*_L_* ^*^	72	100	Ant orbital G*_R_*	70	96
Fusiform G*_L_*	72	100	Fusiform G*_R_*	66	100
Thalamus*_L_*	69	100	Amygdala*_R_*	66	100
Cerebellum*_L_*	66	100	Cerebellum*_R_*	62	100
Parahippoc G*_L_*	63	100	Subcallosal A*_R_*	62	100
Med orbital G*_L_*	63	100	Ant tmp L_R_*	62	100

Subscript L/R: Left/Right. A: area, G: gyrus, L: lobe, Ant: anterior, lat: lateral, med: medial, parahippoc: parahippocamapl, tmp: temporal, Ant tmp L*: Anterior temporal lobe including lateral and medial part.

**Table 3:** Top structures ranked by ability to distinguish TLE-N patients from controls.

Structure	TLE-N_L Individual Sensitivity(%)	Combined Sensitivity(%)	Structure	TLE-N_R Individual Sensitivity(%)	Combined Sensitivity(%)
Substantia nigra*_L_*	72	–	Ant tmp L*_R_*	77	–
Ant orbital G*_L_*	63	72	Ant orbital G*_R_*	77	77
Straight gyrus*_R_*	63	72	Med front G*_R_*	66	77
Med orbital G*_L_*	63	81	Subgenual fr C*_L_*	66	77
Subgenual fr C*_L_*	63	81	Substantia nigra*_R_*	66	88
Lingual G*_R_*	54	81	Straight gyrus*_R_*	66	88
Ant tmp L lat*_L_*	54	90	Inf lat parietal L*_R_*	55	88
Subcallosal A*_R_*	54	90	Ant orbital G*_L_*	55	88
Amygdala*_L_*	54	90	Cerebellum*_R_*	55	88
Cerebellum*_L_*	54	90	Lingual G*_R_*	55	88

Subscript L/R: Left/Right. A: area, C: cortex, G: gyrus, L: lobe, Ant: anterior, fr: frontal, inf: inferior, lat: lateral, med: medial, tmp: temporal.

Hippocampus (right and left) were the most discriminative structures to define the lateralization of the epileptogenic zone in the TLE-HA group, sufficient to achieve correct classification in 98% (one patient with TLE-HA_R was not correctly lateralized using hippocampal volumes alone, with right/left hippocampal volumes of 1610/1586 mm^3^). By adding the volumes of the parahippocampal gyrus to the hippocampal volumes, 100% lateralization accuracy was achieved.

The eight top-ranked structures for identifying the side of the seizure focus in the TLE-N group were: anterior temporal lobe (middle part, right), anterior temporal lobe (lateral part, left), lingual gyrus (right), substantia nigra (right and left), caudate nucleus (left), middle frontal gyrus (left) and nucleus accumbens (left). The lateralization accuracy achieved with this ensemble was 94%.

### Classification Accuracy

The results of the 10-fold cross validation of the various experiments using two different classification procedures along with the optimal number of structures presented to each classification scheme are reported in Table 4. The most important results in 4 are the correct classification rate for Experiment 4 (TLE-N vs controls, 91 ± 3%) and the correct lateralization rate for Experiment 5 (TLE-N patients, 94 ± 4%).

**Table 4:** Sensitivity (Sens), specificity (Spec), accuracy rate (Rate) (all as percentages) and number of selected structures (D) for different experiments. Key results are shown in bold typeface.

Experiment	D	Volumetric Spec	Sens	Rate	D	Spectral Clustering Spec	Sens	Rate
TLE-HA vs. Control	6	93	100	96	10	93	100	96
TLE-HA_R vs. TLE-HA_L	4	100	100	100	4	100	100	100
TLE-HA vs. TLE-N	11	80	98	93	12	93	96	96
TLE-N *vs*. Control	10	97	70	86	10	94	87	**91**
TLE-N_R vs. TLE-N_L	17	84	87	85	8	98	88	**94**

**Figure 5:**
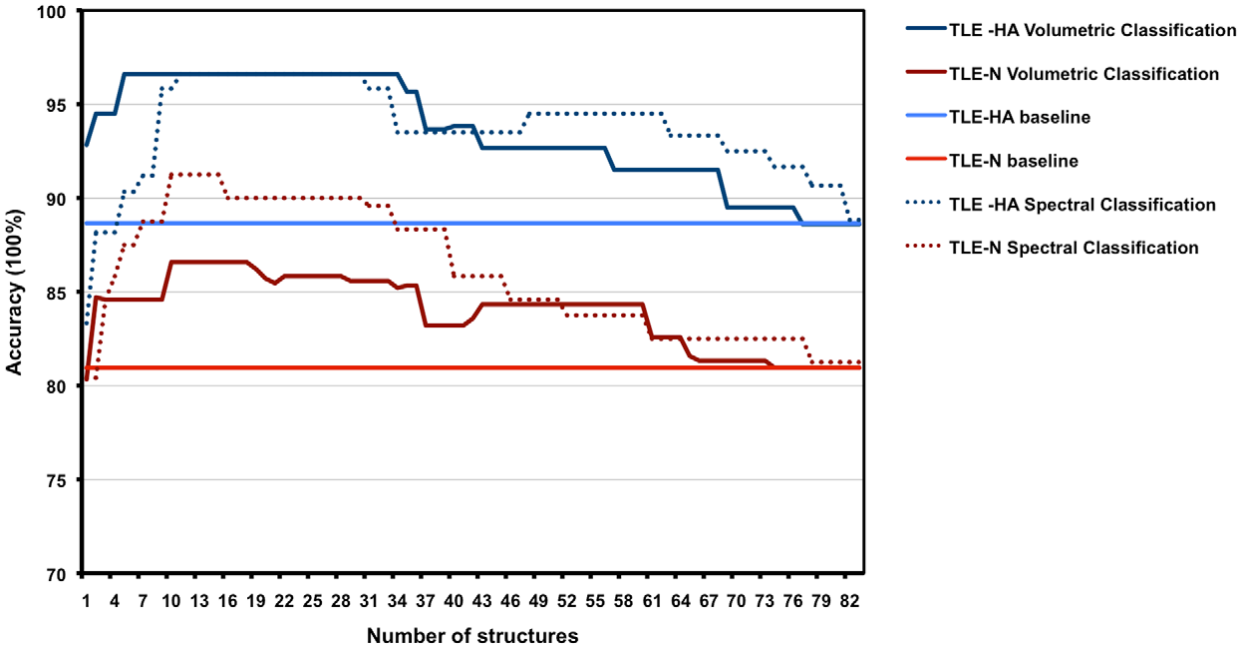
Model response curves for Experiment 1 and 4 for two classification schemes. The classifier accuracy was presented using 83 ranked structures, for each classification experiment (baseline case).

A response curve of model accuracy of the 10-fold cross validation was built based on the total number of structures included in the classification procedures for Experiment 1 and 4 (Figure 5). When the full feature set was input to the SVM (baseline case) for separating TLE-HA group from controls, the overall accuracy was 89%. As shown in Figure 5, choosing the six and ten top-ranked structures yielded the best average correct classification rate for distinguishing TLE-HA subjects from controls, using classification based on structural volumes and spectral analysis, respectively. With attribute selection we reached accuracy levels (96 ± 2%) with only 6–10 features out of 83. In the case of distinguishing the TLE-N group from controls, baseline accuracy was 81% (all-features case). Figure 5 shows that aggregation of the 10 top-ranked structures resulted in the best classification rate when using spectral analysis for separating TLE-N subjects from controls.

Table 5 also shows discrimination results obtained using permutation tests on different classification procedures. The classification rates obtained using morphological similarity (spectral analysis) show greater significance.

**Table 5:** T-statistic and P-value results of the permutation test based on different approaches, Volumetric study and Spectral clustering.

Experiment	Volumetric		Spectral Clustering	
	T-statistic	p-value	T-statistic	p-value
TLE-HA vs. Control	5.4381	< 10^−8^	6.0353	< 10^−8^
TLE-HA vs. TLE-N	2.3824	0.0063	4.7691	< 10^−6^
TLE-N vs. Control	2.4071	0.0153	3.7025	0.0012
TLE-HA_R vs. TLE-HA_L	3.4071	< 10^−9^	3.1005	< 10^−9^
TLE-N_R vs. TLE-N_L	2.8071	0.0115	1.1055	0.0011

When Group 4 (atlases) and Group 5 were combined in a single data-set as the test group to evaluate the classifier trained using Group 1 and 3, 100% of patients were correctly assigned to the appropriate group and 96% of atlases were assigned to the control group.

## Discussion

For many neurological diseases, including TLE, the traditional approach for computer-aided diagnosis focuses on analyzing single structures, such as the hippocampus. The hippocampus is a critical structure of the human limbic system involved in learning and memory processing. In a recent study, Hammers et al. [5] used an automated method for segmenting the hippocampus and detecting hippocampal atrophy in nine subjects with TLE-HA. The method showed high sensitivity, specificity, test-retest reliability, and strong convergence between the automated segmentation and manual tracings of the hippocampus. However, this single structure volumetry approach relies on the presence of HA for diagnosing TLE and would not be applicable in TLE subjects whose MR images appear normal. Other studies of TLE also illustrated that damage and volume loss are not confined to the hippocampus, but involve the amygdala and parahippocampal regions, and often extend to extratemporal cortical regions and subcortical structures as well [27], [68]–[70]. Changes in regions beyond the hippocampus are subtle and complex and are not easily detectable with standard MRI techniques. To our knowledge, this is the first study that uses morphometry of regions covering the entire brain in order to attempt classification of TLE patients and healthy controls.

We employed an automated anatomical segmentation method (MAPER) to delineate 83 structures on MR images of patients diagnosed with TLE and a group of healthy control subjects. The target images for the MAPER segmentations had been acquired at 3T, while the atlas images were 1.5T, raising the question of bias due to the field strength difference. We demonstrated that the method yields equivalent segmentations independent of the field strength of the target image.

The distinction between TLE-HA and TLE-N was based on the established routine diagnostic procedure [52], [53]. This procedure consists of visual analysis of all available imaging by very experienced experts, but does not include routine manual volumetry or routine T2 measurement which are only performed in case of doubt. While we therefore cannot provide these data on an individual basis, our work demonstrates that automatic, quantitative analysis yields clinically relevant information over and above that from the routine approach. The predecessor method for multi-atlas propagation and label fusion [25] was able to correctly identify hippocampal atrophy as part of unilateral HS [5], and Ð importantly Ð correctly identify contralateral hippocampi as being of normal volume. With the current method, better suited to the automatical segmentation of pathological MRIs [49], [51], we replicate the important finding of presumably non-epileptogenic hippocampi being correctly identified as volumetrically normal (see Figure 4), further corroborating the TLE-N/TLE-HA diagnosis by expert consensus.

Another potential limitation of our study is the lack of histopathological findings and surgical outcomes. However, the syndromic distinction between TLE-HA and TLE-N has been demonstrated repeatedly (e.g. [71], [72]) and is replicated by our classification; the lateralization of the epileptogenic side is clearcut in unilateral TLE-HA cases and 100% replicated by our classification; and the veracity of the lateralization in TLE-N patients supported by the excellent lateralization results with the automatic method. While seizure-free outcome following surgery is the ultimate gold standard, we do not think that this standard of proof is necessary for the present study.

A limitation of our study is the risk of overfitting due to the small size of the TLE-N group. The problem has been discussed previously in the context of machine learning from medical imaging data, e.g. [73]. Hua et al. [74] compared different classification methods, examining the relationship between feature numbers and sample size. They describe the peaking phenomenon as a manifestation of overfitting: at first, the classification accuracy increases as more features are added, but decreases once a critical number is surpassed. Hua et al. found that SVM was relatively robust against this phenomenon, compared to, e.g., linear discriminant analysis.

We propose two classification methodologies. Both use structure selection using a kernel-based class separability criterion and rank the most relevant of 83 regions. Our results indicate that the selected regions are sufficient to discriminate between different groups of subjects. The first classification scheme is based on the structural volumes and a support vector machine (SVM-RBF) used to distinguish different group of TLE subjects in this study (TLE-HA, TLE-N) from controls and from each other, and on lateralization of seizure focus. The second approach uses the selected structures to produce indicator features based on morphological similarity information. The linear SVM is then applied to the resulting features. TLE-N patients with absent or weak electroclinical lateralizing features pose an important clinical problem. The ability of the proposed methods to correctly identify the side of seizure onset in the vast majority of TLE-N patients (94%) is clinically promising, potentially reducing the need for invasive intracranial exploration. We conclude that the combination of spectral analysis and a linear SVM yields higher accuracy for discriminating healthy subjects from patients than RBF-based SVMs.

In our study, the overall accuracy of separation of patients with hippocampal atrophy ipsilateral to the seizure focus (TLE-HA) from controls was 96% in both classification schemes. Mainly structures ipsilateral to the epileptogenic side appeared to distinguish patients from controls, with most of these structures are located in the temporal and frontal lobes. The most relevant structures including the ipsilateral hippocampus, parahippocampal gyrus, amygdala, anterior temporal lobe (lateral and medial part), orbital gyrus, thalamus and cerebellum. These results are consistent with those of previous studies of patients with TLE [13], [27], [68]. The sensitivity of the method for detecting HA was 100%, replicating and expanding our earlier findings [5] and suggesting its suitability as a screening tool. To evaluate the proposed method on an independent dataset, we used the group of nine subjects with TLE-HA previously described [5] and the 30 subjects on whose MRIs the original atlases were based, all scanned at 1.5T. All nine TLA-HA were correctly assigned to the patient group, and correctly lateralized, and 29 out of 30 control subjects were correctly assigned.

Hippocampal volume reduction is typically the most relevant measure of lateralization, as it is strongly associated with an ipsilateral seizure focus. The results we obtained are comparable or better than previously described classification methods based on MR images. For example, the accuracy of lateralization in TLE-HA patients is reported 80% in [75] or 90% when including structures other than the hippocampus [75], [76]. Our classification method identified the side of the seizure focus in the TLE-HA group with 100% accuracy using the volumes of hippocampus and parahippocampal gyri. A classification accuracy of 94% was achieved in lateralization of the seizure focus in the TLE-N group based on spectral analysis using volume difference and SVM.

Duchesne et al. [38] reported a maximum of 100% accuracy for lateralization via T1-weighted MR signal intensity and registration metrics in a cuboid-shaped ROI centred on the temporal lobes. This result could be taken to indicate that most of the relevant information is contained in the temporal lobes. However, by taking the whole brain into account we were able to additionally distinguish TLE-N patients from controls with high accuracy (91%). McDonald et al. [17] performed a linear discriminant function analysis to distinguish TLE-HA patients from controls based on hippocampal volumetry, hippocampal asymmetry and a volumetric combination measure that considers right hippocampus, left hippocampus, left amygdala, and left thalamic volumes. They achieved their best results using the combination measure, with accuracy rates of 90% (100% of the controls, 82% of the TLE-HA). They also correctly identified the side of the seizure focus in 91% of the TLE-HA patients. A recent atlas selection method based on greyscale similarity in a dilated hippocampal ROI [8] achieved much lower lateralization accuracy (74%), as expected for a single-atlas method [25] and a mixed cohort of TLE-HA and TLE-N. Other automatic hippocampal segmentation methods have been developed in the fields of epilepsy and dementia. Some have good or excellent segmentation performance even on severely atrophic hippocampi, e.g. [32], [33], [77]–[80]. A recent study using grey matter based segmentation, mean diffusivity and SVM achieved classification of TLE-HA patients from contrls (accuracies of 90–97%) and lateralization (accuracy up to 100%) [81]. These methods are not, however, geared for the specific challenges posed in the diagnosis and lateralization of TLE-N. Most structures highlighted as important for classification in TLE-HA replicate previous results; the main contribution of the present paper as far as TLE-HA is concerned is the successful machine learning classification.

Most of the structures automatically selected for TLE-N classification by the method have face validity. For example, the structures in Table 3 are mostly ipsilateral to the presumed seizure focus, and largely orbitofronto-temporal, with the orbitofrontal region densely connected to the anterior temporal lobe via the uncinate fasciculus. One structure the importance of which for automatic classification is at first glance surprising is the ipsilateral substantia nigra. We, therefore, checked the segmentation of this region visually, but found no obvious segmentation errors. Even if the difference we observe between groups was attributable to a segmentation error, this error would have to occur in one group more than in another, which is unlikely given the acquisition on the same scanner with identical protocols, and also would not explain the importance for lateralisation. Pathophysiologically, smaller substantia nigra volumes might suggest a diminished function of the dopaminergic system. This finding integrates well with established findings on dopamine modulation of seizure activity [82], as well as recent results showing dopaminergic deficits using PET in a number of syndromes (e.g. [83]–[87]) including experimental TLE [88] and clinical TLE [89]. We are thus showing that automatic image analysis using atlas-based segmentation reveals systematic findings that are not observed on visual review of MR images, or with other study designs like voxel-based morphometry, and that such findings may be clinically exploitable.

SVM classifiers are binary by design. The classification problems studied here could be reconsidered as a single multi-class classification problem. However, the aim of this work has not been to introduce a novel classification approach, but instead to use a simple feature combination approach with a readily available classifier to demonstrate the utility of automatic segmentation and structure selection for improving classification between two pairs of diagnostic groups, including clinically relevant distinctions like right-sided versus left-sided TLE-N. A full consideration of multi-class classification (which classifies cases into normal and TLE with type and lateralization information) would be an interesting area of future research.

We performed an automatic segmentation technique and classification method on patients with TLE as a test case for the proposed methodology. Clearly, for other diseases characterized by morphological changes in the brain, pathomorphological features may be detected with this approach. The proposed automated segmentation and classification methodology of MRIs of TLE patients is sufficiently accurate and robust to warrant further exploration of its utility. The techniques await validation on multicentre data, extension to patients with epilepsy other than TLE, and routine clinical application at the individual patient level.

## Supporting Information

Text S1 A full list of ROIs created by MAPER.
(DOC)

Text S2 Mathematical expressions of the kernel-based class separability method for the structure selection.
(DOC)

Text S3 Mathematical expression of the spectral clustering approach.
(DOC)

Text S4 Correlation of the selected brain structures with age.
(DOC)

Text S5 The coefficients of variation for all brain regions created by MAPER of different groups.
(DOC)

## References

[1] Engel J J (1996). Surgery for seizures.. N Engl J Med.

[2] Wiebe S, Blume WT, Girvin JP, Eliasziw M, Effectiveness (2001). A randomized, controlled trial of surgery for temporal-lobe epilepsy.. N Engl J Med.

[3] Cascino GD, Jack J C R, Parisi JE, Sharbrough FW, Hirschorn KA (1991). Magnetic resonance imaging-based volume studies in temporal lobe epilepsy: pathological correlations.. Ann Neurol.

[4] Paesschen WV, Revesz T, Duncan JS, King MD, Connelly A (1997). Quantitative neuropathology and quantitative magnetic resonance imaging of the hippocampus in temporal lobe epilepsy.. Ann Neurol.

[5] Hammers A, Heckemann R, Koepp MJ, Duncan JS, Hajnal JV (2007). Automatic detection and quantification of hippocampal atrophy on MRI in temporal lobe epilepsy: a proof-of-principle study.. Neuroimage.

[6] Bonilha L, Halford JJ, Rorden C, Roberts DR, Rumboldt Z (2009). Automated MRI analysis for identification of hippocampal atrophy in temporal lobe epilepsy.. Epilepsia.

[7] Webb J, Guimond A, Eldridge P, Chadwick D, Meunier J (1999). Automatic detection of hippocampal atrophy on magnetic resonance images.. Magn Reson Imaging.

[8] Akhondi-Asl A, Jafari-Khouzani K, Elisevich K, Soltanian-Zadeh H (2011). Hippocampal volumetry for lateralization of temporal lobe epilepsy: automated versus manual methods.. Neuroimage.

[9] Bernasconi A, Bernasconi N, Caramanos Z, Reutens DC, Andermann F (2000). T2 relax-ometry can lateralize mesial temporal lobe epilepsy in patients with normal MRI.. Neuroimage.

[10] Kobayashi E, D’Agostino MD, Lopes-Cendes I, Berkovic SF, Li ML (2003). Hippocampal atrophy and T2-weighted signal changes in familial mesial temporal lobe epilepsy.. Neurology.

[11] Wieshmann UC, Free SL, Everitt AD, Bartlett PA, Barker GJ (1996). Magnetic resonance imaging in epilepsy with a fast FLAIR sequence.. J Neurol Neurosurg Psychiatry.

[12] Hajnal JV, Bryant DJ, Kasuboski L, Pattany PM, Coene BD (1992). Use of uid attenuated inversion recovery (FLAIR) pulse sequences in MRI of the brain.. J Comput Assist Tomogr.

[13] DeCarli C, Hatta J, Fazilat S, Gaillard WD, Theodore WH (1998). Extratemporal atrophy in patients with complex partial seizures of left temporal origin.. Ann Neurol.

[14] Natsume J, Bernasconi N, Andermann F, Bernasconi A (2003). MRI volumetry of the thalamus in temporal, extratemporal, and idiopathic generalized epilepsy.. Neurology.

[15] Dreifuss S, Vingerhoets FJ, Lazeyras F, Andino SG, Spinelli L (2001). Volumetric measurements of subcortical nuclei in patients with temporal lobe epilepsy.. Neurology.

[16] Margerison JH, Corsellis JA (1966). Epilepsy and the temporal lobes. A clinical, electroencephalo-graphic and neuropathological study of the brain in epilepsy, with particular reference to the temporal lobes.. Brain.

[17] McDonald CR, Hagler DJ, Ahmadi ME, Tecoma E, Iragui V (2008). Subcortical and cerebellar atrophy in mesial temporal lobe epilepsy revealed by automatic segmentation.. Epilepsy Res.

[18] Chupin M, Mukuna-Bantumbakulu AR, Hasboun D, Bardinet E, Baillet S (2007). Anatomically constrained region deformation for the automated segmentation of the hippocampus and the amygdala: Method and validation on controls and patients with Alzheimer’s disease.. Neuroimage.

[19] Freeborough PA, Fox NC, Kitney RI (1997). Interactive algorithms for the segmentation and quantitation of 3-D MRI brain scans.. Comput Methods Programs Biomed.

[20] Shen D, Moffat S, Resnick SM, Davatzikos C (2002). Measuring size and shape of the hippocampus in MR images using a deformable shape model.. Neuroimage.

[21] Bernasconi N, Duchesne S, Janke A, Lerch J, Collins DL (2004). Whole-brain voxel-based statistical analysis of gray matter and white matter in temporal lobe epilepsy.. Neuroimage.

[22] Eriksson SH, Thom M, Symms MR, Focke NK, Martinian L (2009). Cortical neuronal loss and hippocampal sclerosis are not detected by voxel-based morphometry in individual epilepsy surgery patients.. Hum Brain Mapp.

[23] Kelemen A, Szekely G, Gerig G (1999). Elastic model-based segmentation of 3-D neuroradiological data sets.. IEEE Trans Med Imaging.

[24] Barnes J, Boyes RG, Lewis EB, Schott JM, Frost C (2007). Automatic calculation of hip-pocampal atrophy rates using a hippocampal template and the boundary shift integral.. Neurobiol Aging.

[25] Heckemann RA, Hajnal JV, Aljabar P, Rueckert D, Hammers A (2006). Automatic anatomical brain MRI segmentation combining label propagation and decision fusion.. Neuroimage.

[26] Ashburner J, Friston KJ (2000). Voxel-based morphometry–the methods.. Neuroimage.

[27] Labate A, Cerasa A, Gambardella A, Aguglia U, Quattrone A (2008). Hippocampal and thalamic atrophy in mild temporal lobe epilepsy: a VBM study.. Neurology.

[28] Woermann FG, Free SL, Koepp MJ, Sisodiya SM, Duncan JS (1999). Abnormal cerebral structure in juvenile myoclonic epilepsy demonstrated with voxel-based analysis of MRI.. Brain.

[29] Woermann FG, Free SL, Koepp MJ, Ashburner J, Duncan JS (1999). Voxel-by-voxel comparison of automatically segmented cerebral gray matter–a rater-independent comparison of structural MRI in patients with epilepsy.. Neuroimage.

[30] Mehta S, Grabowski TJ, Trivedi Y, Damasio H (2003). Evaluation of voxel-based morphometry for focal lesion detection in individuals.. Neuroimage.

[31] Rohlfing T, Brandt R, Menzel R, Maurer CR (2004). Evaluation of atlas selection strategies for atlas-based image segmentation with application to confocal microscopy images of bee brains.. Neuroimage.

[32] van der Lijn F, den Heijer T, Breteler MMB, Niessen WJ (2008). Hippocampus segmentation in MR images using atlas registration, voxel classification, and graph cuts.. Neuroimage.

[33] Wolz R, Aljabar P, Hajnal JV, Hammers A, Rueckert D (2010). LEAP: learning embeddings for atlas propagation.. Neuroimage.

[34] Connelly A, Paesschen WV, Porter DA, Johnson CL, Duncan JS (1998). Proton magnetic resonance spectroscopy in MRI-negative temporal lobe epilepsy.. Neurology.

[35] Carne RP, O’Brien TJ, Kilpatrick CJ, Macgregor LR, Litewka L (2007). ‘MRI-negative PET-positive’ temporal lobe epilepsy (TLE) and mesial TLE differ with quantitative MRI and PET: a case control study.. BMC Neurol.

[36] Hammers A, Koepp MJ, Hurlemann R, Thom M, Richardson MP (2002). Abnormalities of grey and white matter [11C]umazenil binding in temporal lobe epilepsy with normal MRI.. Brain.

[37] Jutila L, Ylinen A, Partanen K, Alafuzoff I, Mervaala E (2001). MR volumetry of the entorhinal, perirhinal, and temporopolar cortices in drug-refractory temporal lobe epilepsy.. AJNR Am J Neuroradiol.

[38] Duchesne S, Bernasconi N, Bernasconi A, Collins DL (2006). MR-based neurological disease classification methodology: application to lateralization of seizure focus in temporal lobe epilepsy.. Neuroimage.

[39] Duzel E, Kaufmann J, Guderian S, Szentkuti A, Schott B (2004). Measures of hippocampal volumes, diffusion and 1 H MRS metabolic abnormalities in temporal lobe epilepsy provide partially complementary information.. Eur J Neurol.

[40] Uijl SG, Leijten FSS, Arends JBAM, Parra J, van Huffelen AC (2007). The added value of [18F]-uoro-D-deoxyglucose positron emission tomography in screening for temporal lobe epilepsy surgery.. Epilepsia.

[41] Hammers A, Koepp MJ, Free SL, Brett M, Richardson MP (2002). Implementation and application of a brain template for multiple volumes of interest.. Hum Brain Mapp.

[42] Didelot A, Ryvlin P, Lothe A, Merlet I, Hammers A (2008). PET imaging of brain 5-HT1A receptors in the preoperative evaluation of temporal lobe epilepsy.. Brain.

[43] Debets RM, Sadzot B, van Isselt JW, Brekelmans GJ, Meiners LC (1997). Is 11C-umazenil PET superior to 18FDG PET and 123I-iomazenil SPECT in presurgical evaluation of temporal lobe epilepsy?. J Neurol Neurosurg Psychiatry.

[44] Hammers A (2004). Flumazenil positron emission tomography and other ligands for functional imaging.. Neuroimaging Clin N Am.

[45] Hamer H, Morris H, Mascha E, Karafa M, Bingaman W (2002). Complications of invasive video-EEG monitoring with subdural grid electrodes.. Neurology.

[46] Fan Y, Shen D, Davatzikos C (2005). Classification of structural images via high-dimensional image warping, robust feature extraction, and SVM.. Med Image Comput Comput-Assist Interv Int Conf.

[47] Duchesne S, Caroli A, Geroldi C, Barillot C, Frisoni GB (2008). MRI-based automated computer classification of probable AD versus normal controls.. IEEE Trans Med Imaging.

[48] Kloppel S, Stonnington CM, Chu C, Draganski B, Scahill RI (2008). Automatic classification of MR scans in Alzheimer’s disease.. Brain.

[49] Heckemann RA, Keihaninejad S, Aljabar P, Rueckert D, Hajnal JV (2010). Improving intersubject image registration using tissue-class information benefits robustness and accuracy of multi-atlas based anatomical segmentation.. Neuroimage.

[50] Hammers A, Allom R, Koepp MJ, Free SL, Myers R (2003). Three-dimensional maximum probability atlas of the human brain, with particular reference to the temporal lobe.. Hum Brain Mapp.

[51] Heckemann RA, Keihaninejad S, Aljabar P, Gray KR, Nielsen C (2011). Automatic mor-phometry in alzheimer’s disease and mild cognitive impairment.. Neuroimage.

[52] de Tisi J, Bell GS, Peacock JL, McEvoy AW, Harkness WFJ (2011). The long-term outcome of adult epilepsy surgery, patterns of seizure remission, and relapse: a cohort study.. Lancet.

[53] Duncan JS (2011). Selecting patients for epilepsy surgery: synthesis of data.. Epilepsy Behav.

[54] Gousias IS, Rueckert D, Heckemann RA, Dyet LE, Boardman JP (2008). Automatic segmentation of brain MRIs of 2-year-olds into 83 regions of interest.. Neuroimage.

[55] Smith SM, Jenkinson M, Woolrich MW, Beckmann CF, Behrens TE (2004). Advances in functional and structural MR image analysis and implementation as FSL.. Neuroimage.

[56] Rueckert D, Sonoda LI, Hayes C, Hill DL, Leach MO (1999). Nonrigid registration using free-form deformations: application to breast MR images.. IEEE Trans Med Imaging.

[57] Kittler J, Hatef M, Duin RPW, Matas J (1998). On combining classifiers.. IEEE Trans Pattern Anal Mach Intell.

[58] Free SL, Bergin PS, Fish DR, Cook MJ, Shorvon SD (1995). Methods for normalization of hippocampal volumes measured with MR.. AJNR Am J Neuroradiol.

[59] Keihaninejad S, Heckemann RA, Fagiolo G, Symms MR, Hajnal JV (2010). A robust method to estimate the intracranial volume across MRI field strengths (1.5T and 3T).. Neuroimage.

[60] Wang L (2008). Feature selection with kernel class separability.. IEEE Trans on Pattern Analysis and Machine Intelligence.

[61] Fukunaga K (1972). Introduction to Statistical Pattern Recognition..

[62] von Luxburg U (2007). A tutorial on spectral clustering.. Statistics and Computing.

[63] Aljabar P, Rueckert D, Crum WR (2008). Automated morphological analysis of magnetic resonance brain imaging using spectral analysis.. Neuroimage.

[64] Belkin M, Niyogi P (2003). Laplacian eigenmaps for dimensionality reduction and data representation.. Neural Computation.

[65] Vapnik VN (1999). An overview of statistical learning theory.. IEEE Trans Neural Network.

[66] Prechelt L (1998). Automatic early stopping using cross validation: quantifying the criteria.. Neural Network.

[67] Nichols TE, Holmes AP (2002). Nonparametric permutation tests for functional neuroimaging: a primer with examples.. Hum Brain Mapp.

[68] Bonilha L, Rorden C, Castellano G, Cendes F, Li LM (2005). Voxel-based morphometry of the thalamus in patients with refractory medial temporal lobe epilepsy.. Neuroimage.

[69] Keller SS, Mackay CE, Barrick TR, Wieshmann UC, Howard MA (2002). Voxel-based mor-phometric comparison of hippocampal and extrahippocampal abnormalities in patients with left and right hippocampal atrophy.. Neuroimage.

[70] Moran NF, Lemieux L, Kitchen ND, Fish DR, Shorvon SD (2001). Extrahippocampal temporal lobe atrophy in temporal lobe epilepsy and mesial temporal sclerosis.. Brain.

[71] Woermann FG, McLean MA, Bartlett PA, Parker GJ, Barker GJ (1999). Short echo time single-voxel 1 h magnetic resonance spectroscopy in magnetic resonance imaging-negative temporal lobe epilepsy: different biochemical profile compared with hippocampal sclerosis.. Ann Neurol.

[72] Carne RP, O’Brien TJ, Kilpatrick CJ, MacGregor LR, Hicks RJ (2004). Mri-negative pet- positive temporal lobe epilepsy: a distinct surgically remediable syndrome.. Brain.

[73] Wang Y, Fan Y, Bhatt P, Davatzikos C (2010). High-dimensional pattern regression using machine learning: from medical images to continuous clinical variables.. Neuroimage.

[74] Hua J, Xiong Z, Lowey J, Suh E, Dougherty ER (2005). Optimal number of features as a function of sample size for various classification rules.. Bioinformatics.

[75] Bernasconi N, Bernasconi A, Caramanos Z, Antel SB, Andermann F (2003). Mesial temporal damage in temporal lobe epilepsy: a volumetric MRI study of the hippocampus, amygdala and parahippocampal region.. Brain.

[76] Cendes F, Andermann F, Gloor P, Evans A, Jones-Gotman M (1993). MRI volumetric measurement of amygdala and hippocampus in temporal lobe epilepsy.. Neurology.

[77] Chupin M, Hammers A, Liu RSN, Colliot O, Burdett J (2009). Automatic segmentation of the hippocampus and the amygdala driven by hybrid constraints: method and validation.. Neuroimage.

[78] Leung KK, Barnes J, Ridgway GR, Bartlett JW, Clarkson MJ (2010). Automated cross-sectional and longitudinal hippocampal volume measurement in mild cognitive impairment and Alzheimer’s disease.. Neuroimage.

[79] Avants BB, Yushkevich P, Pluta J, Minko_ D, Korczykowski M (2010). The optimal template effect in hippocampus studies of diseased populations.. Neuroimage.

[80] Morra JH, Tu Z, Apostolova LG, Green AE, Toga AW (2010). Comparison of adaboost and support vector machines for detecting Alzheimer’s disease through automated hippocampal segmentation.. IEEE Trans Med Imaging.

[81] Focke NK, Yogarajah M, Symms MR, Gruber O, Paulus W (2011). Automated MR image classification in temporal lobe epilepsy..

[82] Deransart C, Vercueil L, Marescaux C, Depaulis A (1998). The role of basal ganglia in the control of generalized absence seizures.. Epilepsy Res.

[83] Ciumas C, Wahlin TBR, Jucaite A, Lindstrom P, Halldin C (2008). Reduced dopamine transporter binding in patients with juvenile myoclonic epilepsy.. Neurology.

[84] Ciumas C, Wahlin TBR, Espino C, Savic I (2010). The dopamine system in idiopathic generalized epilepsies: identification of syndrome-related changes.. Neuroimage.

[85] Landvogt C, Buchholz HG, Bernedo V, Schreckenberger M, Werhahn KJ (2010). Alteration of dopamine d2/d3 receptor binding in patients with juvenile myoclonic epilepsy.. Epilepsia.

[86] Fedi M, Berkovic SF, Scheffer IE, O’Keefe G, Marini C (2008). Reduced striatal d1 receptor binding in autosomal dominant nocturnal frontal lobe epilepsy.. Neurology.

[87] Korja M, Kaasinen V, Lamusuo S, Parkkola R, Någren K (2007). Substantial thalamostriatal dopaminergic defect in unverricht-lundborg disease.. Epilepsia.

[88] Yakushev IY, Dupont E, Buchholz HG, Tillmanns J, Debus F (2010). In vivo imaging of dopamine receptors in a model of temporal lobe epilepsy.. Epilepsia.

[89] Bouilleret V, Semah F, Chassoux F, Mantzaridez M, Biraben A (2008). Basal ganglia in-volvement in temporal lobe epilepsy: a functional and morphologic study.. Neurology.

