# Supplementary material for: Classification and Lateralization of Temporal Lobe Epilepsies with and without Hippocampal Atrophy Based on Whole-Brain Automatic MRI Segmentation
Source: PLoS One. 2012 Apr 16;7(4):e33096. doi: 10.1371/journal.pone.0033096 (PMC3327701; doi:10.1371/journal.pone.0033096)
Supplement: Text S1 — A full list of ROIs created by MAPER. (DOC) [file pone.0033096.s001.doc]

**Classification and lateralization of temporal lobe epilepsies with and**

**without hippocampal atrophy based on whole-brain automatic MRI**

**segmentation**

Shiva Keihaninejad, Rolf A. Heckemann, Ioannis S. Gousias, Joseph

V.Hajnal, John S. Duncan, Paul Aljabar, Daniel Rueckert, Alexander

Hammers

**Supporting Information**

**S.1. A full list of ROIs created by MAPER.**

| **Number in Atlas** | **Name of Structures** | |
| --- | --- | --- |
| ***Temporal Lobe*** |  | |
| 1;2 | Hippocampus | |
| 3;4 | Amygdala | |
| 5;6 | Anterior temporal lobe, medial part | |
| 7;8 | Anterior temporal lobe, lateral part | |
| 9;10 | Parahippocampal and ambient gyri | |
| 11;12 | Superior temporal gyrus, posterior part | |
| 13;14 | Middle and inferior temporal gyrus | |
| 15;16 | Fusiform gyrus | |
| 30;31 | Posterior temporal lobe | |
| 82;83 | Superior temporal gyrus, anterior part | |
| ***Posterior Fossa*** |  | |
| 17;18 | Cerebellum | |
| 19 | Brainstem | |
| ***Insula and Cingulate gyri*** |  | |
| 20;21 | Insula | |
| 24;25 | Cingulate gyrus, anterior part | |
| 26;27 | Gyrus cinguli, posterior part | |
| ***Frontal Lobe*** |  | |
| 28;29 | Middlle frontal gyrus | |
| 50;51 | Precentral gyrus | |
| 54;55 | Anterior orbital gyrus | |
| 56;57 | Inferior frontal gyrus | |
| 58;59 | Superior frontal gyrus | |
| 68;69 | Medial orbital gyrus | |
| 70;71 | Lateral orbital gyrus | |
| 72;73 | Posterior orbital gyrus | |
| 76;77 | Subgenual frontal cortex | |
| 78;79 | Subcallosal area | |
| 80;81 | Pre-subgenual frontal cortex | |
| ***Occipital Lobe*** |  | |
| 64;65 | Lingual gyrus | |
| 66;67 | Cuneus | |
| 22;23 | Lateral remainder of occipital lobe | |
| ***Parietal Lobe*** |  | |
| 52;53 | Straight gyrus | |
| 60;61 | Postcentral gyrus | |
| 62;63 | Superior parietal gyrus | |
| 32;33 | Inferiolateral remainder of parietal lobe | |
| ***Central Structures*** |  | |
| 34;35 | Caudate nucleus | |
| 36;37 | Nucleus accumbens | |
| 38;39 | Putamen | |
| 40;41 | Thalamus | |
| 42;43 | Pallidum | |
| 44 | Corpus callosum | |
| 74;75 | Substantia nigra | |
| ***Ventricles*** |  | |
| 45;46 | Lateral ventricle (excluding temporal horn) | |
| 47;48 | Lateral ventricle, temporal horn | |
| 49 | | Third ventricle |
