# Supplementary material for: Classification and Lateralization of Temporal Lobe Epilepsies with and without Hippocampal Atrophy Based on Whole-Brain Automatic MRI Segmentation
Source: PLoS One. 2012 Apr 16;7(4):e33096. doi: 10.1371/journal.pone.0033096 (PMC3327701; doi:10.1371/journal.pone.0033096)
Supplement: Text S2 — Mathematical expressions of the kernel-based class separability method for the structure selection. (DOC) [file pone.0033096.s002.doc]

**Classification and lateralization of temporal lobe epilepsies with and without hippocampal atrophy based on whole-brain automatic MRI segmentation**

Shiva Keihaninejad, Rolf A. Heckemann, Ioannis S. Gousias, Joseph V.Hajnal, John S. Duncan, Paul Aljabar, Daniel Rueckert, Alexander Hammers

**Supporting Information**

**S.2. Mathematical expressions of the kernel-based class separability method for the structure selection**

The advantage of the kernel-based class separability criterion over more conventional criteria such as the Bhattacharyya distance, Kullback-Leibler divergence, and Matusita distance [60] is that no assumption is made regarding the conditional probability densities of features (volumes of structures).

In a two-class problem, let $c_{i}$ denote the set of features from the $i^{\text{th}}$ class (). is defined as . In addition, $n_{i}$ and $n$ denote the number of samples in $c_{i}$ and $c$, respectively. A simple class separability measure can be derived from the within- and between-class scatter matrices, $S_{W}$ and $S_{B}$, which are defined as:

(1)

where $m_{i}$ is the mean vector associated with the $i^{\text{th}}$ class and $m$ is the mean vector of the entire dataset. A large class separability means small within-class scattering but large between-class scattering. The matrices $S_{W}$ and $S_{B}$ are typically used to derive separation criteria. One example is . In this measure, the matrices are evaluated via the mean and variance of the data. The use of these matrices, however, has an implicit Gaussian assumption. To address this problem, Wang et al. in [59], apply a kernel transform to the data. Let $K$ denote an matrix of the transformed data with and let $K_{\text{A,B}}$ represent the sub-matrix obtained with the constraints and . The class separability measure may then be defined as [59]:

(2)

where the operator $\text{Sum}(.)$ denotes the summation of all elements of a matrix. The kernel based class separability criterion of the feature set $c$ proposed in (Wang, 2008) is:

(3)

$J^{f}$ is dependent on the kernel parameter $q$, and an inaccurate setting of $q$ can reduce the effectiveness of this criterion. To address this problem, the maximum of $J^{f}$ over the kernel parameter $q$ is considered as the class separability criterion, i.e.:

(4)

The maximization of $J_{q}$ over $q$ can be efficiently solved by gradient-based optimization. In BIN, the class separability criterion (Eq. (4)) is individually applied to each of the features and those with the largest values are selected.
