# Supplementary material for: Classification and Lateralization of Temporal Lobe Epilepsies with and without Hippocampal Atrophy Based on Whole-Brain Automatic MRI Segmentation
Source: PLoS One. 2012 Apr 16;7(4):e33096. doi: 10.1371/journal.pone.0033096 (PMC3327701; doi:10.1371/journal.pone.0033096)
Supplement: Text S3 — Mathematical expression of the spectral clustering approach. (DOC) [file pone.0033096.s003.doc]

**Classification and lateralization of temporal lobe epilepsies with and without hippocampal atrophy based on whole-brain automatic MRI segmentation**

Shiva Keihaninejad, Rolf A. Heckemann, Ioannis S. Gousias, Joseph V.Hajnal, John S. Duncan, Paul Aljabar, Daniel Rueckert, Alexander Hammers

**Supporting Information**

**S.3. Mathematical expression of the spectral clustering approach**

A graph of N subjects (nodes) with edges weighted by the similarity between nodes is constructed. The elements of the affinity matrix $W$ are the pairwise similarities of the subjects calculated with a similarity function. The affinity matrix is then used to form the normalized Laplacian matrix $(L)$, where and $D_{\text{ij}}=0$ for . $D$ is a diagonal degree matrix, which measures the total similarity between each subject and all others by summing the edge-weights along each row. Spectral analysis constructs the matrix from the eigenvectors $v_{i}$ corresponding to the first $k$ ordered non-zero eigenvalues of $L$.

In this work, we used the volumetric difference described by the Gaussian similarity function , where $c$ is a constant of value 2 as obtained empirically in [62] and variables $x_{i}$ and $x_{j}$ correspond to the normalized volumes of a particular structure in subjects $i$ and $j$, respectively. The volumes of corresponding selected structures over $N$ subjects were transformed to z-scores, by subtracting the mean and dividing by the standard deviation. Separate Laplacian matrices $L_{1},...\text{,L}_{D}$ are constructed for the $D$ structures identified by structure selection. The feature data from separate Laplacian matrices are then combined to create the feature matrix, with each row corresponding to a feature extracted for a subject. Since ours is a two class problem, we chose $\text{k=}2$ as suggested in [61].
