# Supplementary material for: Classification and Lateralization of Temporal Lobe Epilepsies with and without Hippocampal Atrophy Based on Whole-Brain Automatic MRI Segmentation
Source: PLoS One. 2012 Apr 16;7(4):e33096. doi: 10.1371/journal.pone.0033096 (PMC3327701; doi:10.1371/journal.pone.0033096)
Supplement: Text S4 — Correlation of the selected brain structures with age. (DOC) [file pone.0033096.s004.doc]

| **Structure** | **Patients** | **Control** |
| --- | --- | --- |
| Hippocampus_R | 0.05 | 0.04 |
| Hippocampus_L | -0.23 | -0.20 |
| Amygdala_R | 0.09 | -0.11 |
| Amygdala_L | -0.03 | -0.09 |
| Anterior temporal lobe, medial part_R | 0.02 | 0.00 |
| Anterior temporal lobe, medial part_L | -0.06 | -0.08 |
| Anterior temporal lobe, lateral part_R | -0.00 | -0.05 |
| Anterior temporal lobe, lateral part_L | -0.08 | -0.12 |
| Parahippocampal and ambient gyri_R | 0.10 | 0.14 |
| Parahippocampal and ambient gyri_L | -0.03 | -0.05 |
| Fusiform gyrus_R | 0.04 | -0.08 |
| Fusiform gyrus_L | -0.07 | -0.11 |
| Cerebellum_R | -0.19 | -0.20 |
| Cerebellum_L | -0.17 | -0.19 |
| Middlle frontal gyrus_R | -0.28 | -0.31 |
| Middlle frontal gyrus_L | -0.25 | -0.24 |
| Anterior orbital gyrus_R | -0.08 | -0.05 |
| Anterior orbital gyrus_L | -0.05 | -0.10 |
| Medial orbital gyrus_R | -0.08 | -0.09 |
| Medial orbital gyrus_L | -0.13 | -0.10 |
| Subgenual frontal cortex_R | -0.18 | -0.17 |
| Subgenual frontal cortex_L | -0.21 | -0.20 |
| Subcallosal area_R | 0.20 | 0.19 |
| Subcallosal area_L | 0.18 | 0.13 |
| Lingual gyrus_R | -0.13 | -0.17 |
| Lingual gyrus_L | -0.07 | -0.10 |
| Straight gyrus_R | -0.09 | -0.08 |
| Straight gyrus_L | -0.11 | -0.14 |
| Superior parietal gyrus_R | -0.25 | -0.27 |
| Superior parietal gyrus_L | -0.18 | -0.26 |
| Inferiolateral parietal lobe_R | -0.21 | -0.23 |
| Inferiolateral parietal lobe_L | -0.19 | -0.20 |
| Thalamus_R | -0.20 | -0.18 |
| Thalamus_L | -0.16 | -0.17 |
| Substantia nigra_R | -0.16 | -0.18 |
| Substantia nigra_L | -0.15 | -0.14 |

**Classification and lateralization of temporal lobe epilepsies with and**

**without hippocampal atrophy based on whole-brain automatic MRI**

**segmentation**

Shiva Keihaninejad, Rolf A. Heckemann, Ioannis S. Gousias, Joseph

V.Hajnal, John S. Duncan, Paul Aljabar, Daniel Rueckert, Alexander

Hammers

**Supporting Information**

**S.4. Correlation of brain structural volume with age**

Table shows the correlation coefficient of brain structures, which used in the classification process, with age for patient and control groups based on nonparametric (Spearman’s) correlation. Most of the brain structures showed negative correlation with age while these correlations didn’t reach the p=0.05 level of significance.
