# Supplementary material for: Classification and Lateralization of Temporal Lobe Epilepsies with and without Hippocampal Atrophy Based on Whole-Brain Automatic MRI Segmentation
Source: PLoS One. 2012 Apr 16;7(4):e33096. doi: 10.1371/journal.pone.0033096 (PMC3327701; doi:10.1371/journal.pone.0033096)
Supplement: Text S5 — The coefficients of variation for all brain regions created by MAPER of different groups. (DOC) [file pone.0033096.s005.doc]

**Classification and lateralization of temporal lobe epilepsies with and**

**without hippocampal atrophy based on whole-brain automatic MRI**

**segmentation**

Shiva Keihaninejad, Rolf A. Heckemann, Ioannis S. Gousias, Joseph

V.Hajnal, John S. Duncan, Paul Aljabar, Daniel Rueckert, Alexander

Hammers

**Supporting Information**

**S.5. Coefficient of variation of brain structures**

Table shows the coefficient of variation (CV) of different brain structures for TLE-HA, TLE-N and Control groups. The average CV of left and right structures are reported. The TLE-HA and TLE-N groups both contain the subjects with left and right side of seizure onset.

| **Structure** | **TLE-HA** | **TLE-N** | **Control** |
| --- | --- | --- | --- |
| Hippocampus | 0.21 | 0.08 | 0.08 |
| Amygdala | 0.14 | 0.11 | 0.1 |
| Anterior temporal lobe, medial part | 0.14 | 0.1 | 0.1 |
| Anterior temporal lobe, lateral part | 0.16 | 0.15 | 0.12 |
| Parahippocampal and ambient gyri | 0.12 | 0.1 | 0.1 |
| Superior temporal gyrus, posterior part | 0.12 | 0.09 | 0.11 |
| Middle and inferior temporal gyrus | 0.14 | 0.11 | 0.11 |
| Fusiform gyrus | 0.14 | 0.12 | 0.12 |
| Posterior temporal lobe | 0.03 | 0.06 | 0.05 |
| Superior temporal gyrus, anterior part | 0.16 | 0.11 | 0.13 |
| Cerebellum | 0.16 | 0.09 | 0.05 |
| Brainstem | 0.17 | 0.19 | 0.17 |
| Insula | 0.08 | 0.07 | 0.07 |
| Cingulate gyrus, anterior part | 0.14 | 0.15 | 0.1 |
| Gyrus cinguli, posterior part | 0.1 | 0.1 | 0.1 |
| Middlle frontal gyrus | 0.11 | 0.08 | 0.1 |
| Precentral gyrus | 0.09 | 0.09 | 0.08 |
| Anterior orbital gyrus | 0.15 | 0.12 | 0.12 |
| Inferior frontal gyrus | 0.1 | 0.11 | 0.09 |
| Superior frontal gyrus | 0.09 | 0.06 | 0.09 |
| Medial orbital gyrus | 0.13 | 0.1 | 0.12 |
| Lateral orbital gyrus | 0.11 | 0.11 | 0.12 |
| Posterior orbital gyrus | 0.12 | 0.12 | 0.08 |
| Subgenual frontal cortex | 0.21 | 0.22 | 0.14 |
| Subcallosal area | 0.2 | 0.16 | 0.14 |
| Pre-subgenual frontal cortex | 0.24 | 0.28 | 0.23 |
| Lingual gyrus | 0.13 | 0.1 | 0.09 |
| Cuneus | 0.14 | 0.12 | 0.11 |
| Lateral remainder of occipital lobe | 0.11 | 0.1 | 0.09 |
| Straight gyrus | 0.12 | 0.1 | 0.1 |
| Postcentral gyrus | 0.1 | 0.08 | 0.07 |
| Superior parietal gyrus | 0.1 | 0.1 | 0.08 |
| Inferiolateral remainder of parietal lobe | 0.11 | 0.08 | 0.09 |
| Caudate nucleus | 0.1 | 0.1 | 0.09 |
| Nucleus accumbens | 0.17 | 0.13 | 0.15 |
| Putamen | 0.11 | 0.09 | 0.1 |
| Thalamus | 0.13 | 0.08 | 0.08 |
| Pallidum | 0.16 | 0.11 | 0.15 |
| Corpus callosum | 0.11 | 0.1 | 0.09 |
| Substantia nigra | 0.13 | 0.14 | 0.1 |
| Lateral ventricle (excluding temporal horn) | 0.49 | 0.59 | 0.44 |
| Lateral ventricle, tem | 0.2 | 0.18 | 0.17 |
